# Associations Between Screen Time Use and Health Outcomes Among US Teenagers

**DOI:** 10.5888/pcd22.240537

**Published:** 2025-07-10

**Authors:** Benjamin Zablotsky, Amanda E. Ng, Lindsey I. Black, Gelila Haile, Jonaki Bose, Jessica R. Jones, Stephen J. Blumberg

**Affiliations:** 1 National Center for Health Statistics, Hyattsville, Maryland

## Abstract

**Introduction:**

Associations between screen time and health outcomes among teenagers are well established. However, most studies use parent-reported information, which may misrepresent the magnitude or nature of these associations. In addition, timely nationally representative estimates are needed to correspond with evolving screen use. This study aimed to address these gaps by using data from a nationally representative survey of teenagers.

**Methods:**

Data came from the 2021–2023 National Health Interview Survey-Teen (NHIS-Teen), a follow-back web-based survey designed to collect health information directly from teenagers aged 12 to 17 years. NHIS-Teen provides a unique opportunity to assess teenagers’ self-reported health in conjunction with a rich set of parent-reported covariates, including family income, from the National Health Interview Survey. This study examines associations between high daily non-schoolwork screen time, defined as 4 or more hours of daily screen time, and adverse health outcomes across the domains of physical activity, sleep, weight, mental health, and perceived support.

**Results:**

Teenagers with higher non-schoolwork screen use were more likely to experience a series of adverse health outcomes, including infrequent physical activity, infrequent strength training, being infrequently well-rested, having an irregular sleep routine, weight concerns, depression symptoms, anxiety symptoms, infrequent social and emotional support, and insufficient peer support.

**Conclusion:**

Results of this study include associations between high screen time and poor health among teenagers using self-reported data. Future work may further investigate these associations and their underlying mechanisms, including the content viewed on screens and the interactions taking place across screens.

**Summary**

**What is known on this topic?**

Teenagers with higher non-schoolwork daily screen time are more likely to experience a series of adverse health outcomes.

**What is added by this report?**

Teenagers with higher non-schoolwork screen use were more likely to engage in infrequent physical activity and to have weight concerns, depression symptoms, anxiety symptoms, infrequent social and emotional support, insufficient peer support, and an irregular sleep routine.

**What are the implications for public health practice?**

As the use of screens continues to increase and the ways teenagers interact with their screens diversify, timely estimates of screen use among teenagers are needed to better understand the effect of screens on the health of teenagers, as reported by teenagers.

## Introduction

The availability of smartphones among teenagers (hereinafter, teens) has increased significantly in the past decade, with nearly all teens having access to smartphones or other internet-connected devices as of 2022 (1). Accompanying this increase in availability has been a notable increase in daily screen time, with an additional bump during the COVID-19 pandemic (2). High screen time can displace important health behaviors, such as physical activity and adequate sleep (3,4). Teens with limited physical activity and sleep are at risk for several negative mental health outcomes, including anxiety, depression, and poorer quality of life (5–9). The content viewed on screens may also have a direct impact on a teen’s mental health (10). Previous research has found a connection between screen usage and poorer mental health, including perceptions of social connectedness (11,12).

With teens’ continued screen use, combined with the influence of extensive technology, it remains critical to better understand the patterns of screen time use and health outcomes with timely, nationally representative data. To date, much of the nationally representative epidemiologic research examining the relationships between teen screen time and health has relied on parent report, with few, like the Youth Risk Behavior Survey (YRBS) (13), using teen self-report. Data obtained directly from teens may provide additional insights when compared with parent-reported data, particularly as related to the teen’s internal world, mental health struggles, or perceived social and emotional supports (14). The National Health Interview Survey-Teen (NHIS-Teen) has the additional benefit of being linkable to family-level social determinants of health reported by their parents as part of the National Health Interview Survey (NHIS) (eg, family income, food security).

The objective of this study was to examine the relationship between daily screen time use among teens and several health outcomes, including physical activity, sleep, weight, mental health, and perceived support. Significant teen, family, and geographic differences in screen time use were identified in a recent study (15), including higher use among older teens, teens in metropolitan areas, and those from less educated families. These sociodemographic characteristics were controlled for to better understand the relationship between screen use and health-related outcomes. It was hypothesized that after adjustment for sociodemographic characteristics, teens with 4 or more hours of daily screen time would have poorer health outcomes than those with less than 4 hours of daily screen time across several health domains.

## Methods

### Data source

Data used in this report come from NHIS-Teen, a follow-back, self-administered, web-based survey of teens aged 12 to 17 years. Teens who participated in NHIS-Teen had parents who completed the NHIS Sample Child interview and provided permission for their teen to participate in NHIS-Teen. Topic areas in NHIS-Teen include doctor visits, sleep, physical activity and screen time, mental health, social and emotional supports, and experiences with bullying and discrimination. The parent permission rate for NHIS-Teen was 60.4% and the teen participation rate was 46.2%, resulting in an overall NHIS-Teen interview rate of 27.9%. Detailed information about NHIS-Teen, including survey methods, weighting, and the questionnaire is available at www.cdc.gov/nchs/nhis/teen. 

A total of 1,958 teens completed NHIS-Teen between July 2021 and December 2023. Teens with missing data for screen time were excluded, resulting in a total analytic sample of 1,952. 

### Measures

#### Screen time

Daily screen time was measured using the question, “On most weekdays, how many hours do you spend a day in front of a TV, computer, cell phone, or other electronic device, watching programs, playing games, accessing the internet, or using social media?” Teens were given the options of “less than 1 hour,” “1 hour,” “2 hours,” “3 hours,” and “4 or more hours.” Teens were told as part of the question’s instructional text to exclude time spent doing schoolwork. For this analysis, teens reporting “4 or more hours” were categorized as having high daily screen time. This was selected to effectively cut the sample in half while categorizing the highest screen time users.

#### Physical activity

*Infrequent physical activity.* Teens were asked, “In a typical week during the school year, how often do you exercise, play a sport, or participate in physical activity for at least 60 minutes a day?” — mirrored after the Centers for Disease Control and Prevention’s *Physical Activity Guidelines for Americans, 2nd edition* (16). Response options were “never,” “some days,” “most days,” and “every day.” Teens were considered to have infrequent physical activity if they answered “never” or “some days.”

*Infrequent muscle strengthening.* Teens were asked, “In a typical week during the school year, how often do you do exercises to strengthen or tone your muscles, such as sit-ups, push-ups, or weightlifting?” Response options were “never,” “some days,” “most days,” and “every day.” Teens were considered to have infrequent muscle strengthening if they answered “never.”

#### Sleep

*Infrequently well-rested.* Teens were asked, “In a typical week during the school year, how often do you wake up well-rested?” Response options were “never,” “some days,” “most days,” and “every day.” Teens who selected “never” and “some days” were considered to be infrequently well-rested.

*Irregular sleep routine.* Teens were also asked whether they had a regular bedtime (“In a typical week during the school year, on nights you have school the next day, how often do you go to bed at the same time?”) and waketime (“In a typical week during the school year, on school days, how often do you wake up at the same time?”) Teens were given the same response options of “never,” “some days,” “most days,” and “every day” for each question, and teens who did not answer “most days” and “every day” for both questions were considered to have an irregular sleep routine.

#### Weight concern

Teens were asked “Are you concerned about your weight?” with the response options of “yes, it’s too high,” “yes, it’s too low,” and “no.” Teens who said their weight was too high or too low were considered to have weight concerns.

#### Mental health

*Anxiety symptoms.* NHIS-Teen included the Generalized Anxiety Disorder-2 (GAD-2) scale (17) which consists of 2 questions: “Over the last 2 weeks, how often have you been bothered by feeling nervous, anxious, or on edge?” and “Over the last 2 weeks, how often have you been bothered by not being able to stop or control worrying?” Response options included “not at all,” “several days,” “more than half the days,” and “nearly every day,” with the responses scored 0, 1, 2, or 3, respectively. Teens were required to answer both questions to have a total score calculated, and teens who scored 3 or more on the combined questions were considered to have anxiety symptoms per GAD-2 scoring guidelines.

*Depression symptoms.* Teens also completed the Patient Health Questionnaire-2 (PHQ-2) scale (18). This scale also consisted of 2 questions: “Over the last 2 weeks, how often have you been bothered by having little interest or pleasure in doing things?” and “Over the last 2 weeks, how often have you been bothered by feeling down, depressed, or hopeless?” Like the GAD-2, response options were “not at all,” “several days,” “more than half the days,” and “nearly every day,” with the same assigned value per response. Teens were required to answer both questions to have a total score calculated, and teens who scored 3 or more on the combined questions were considered to have depression symptoms per PHQ-2 scoring guidelines.

#### Perceived support

*Insufficient peer support.* Teens were asked 2 questions about the peer support in their lives: “How much can you rely on your friends for help if you have a serious problem?” and “How much can you open up to your friends if you need to talk about your worries?” Both questions had the response options of “a lot,” “some,” “a little,” and “not at all.” Teens who did not answer “a lot” or “some” to both questions were considered to have insufficient peer support. Teens who did not answer both questions were not included in the outcome.

*Infrequent social and emotional support.* Teens were asked “How often do you get the social and emotional support you need?” with the response options of “always,” “usually,” “sometimes,” “rarely,” and “never.” Teens who answered “sometimes,” “rarely,” or “never” were considered to have infrequent social and emotional support.

Sociodemographic characteristics were reported by parents as part of the NHIS Sample Child interview and included teen sex; age (12–14 y, 15–17 y); race and Hispanic origin (non-Hispanic Asian, non-Hispanic Black, non-Hispanic White, Hispanic); urbanization level (metropolitan area [large central, large fringe, medium, and small] or nonmetropolitan area [micropolitan and noncore counties]) (19); highest level of education of any resident parent (some college or associate’s degree or less or bachelor’s degree or higher); and family income as a percentage of federal poverty level (less than 200%, 200% or more) (NHIS income data includes imputation when missing) (20–22). As reported elsewhere (15) based on analyses of the same dataset, older teens, non-Hispanic Black teens, and teens with less educated parents were more likely to have 4 or more hours of daily screen time. No differences were seen by sex, family income, or urbanization level. The supplemental table (Appendix) explores the percentage distribution of sociodemographic characteristics by screen time among teens, comparing those with less than 4 hours and those with 4 or more hours of daily screen time.

### Statistical analysis

The prevalence of various health outcomes was examined comparing teens with 4 or more hours of daily screen time to those with less than 4 hours of daily screen time. Differences between groups were calculated using bivariate Poisson regressions (calculated as prevalence ratios); multivariable Poisson regressions were conducted to determine whether these differences remained after adjustment for sociodemographic characteristics including teen age, sex, race and Hispanic origin, urbanization level, highest educated parent, and family income. A sensitivity analysis included adding interactions between screen time and sex and screen time and age to the final models.

All estimates were weighted, and confidence intervals accounted for the complex sample design of the NHIS-Teen (23) using Stata SE version 17.0 (StataCorp LLC), thereby allowing for nationally representative estimates, accounting for nonresponse both for parent permission and teen participation. All estimates in this report met National Center for Health Statistics data presentation standards for proportions (24). Missingness on health outcomes of interest ranged from 0.1% to 3.8%.

## Results

**Figure 1:**
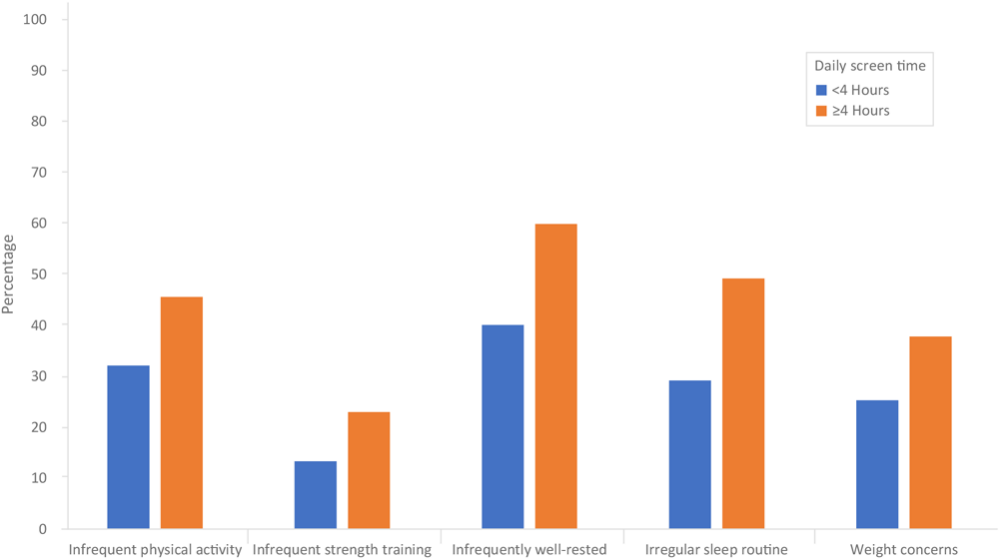
Prevalence of physical activity, sleep, and weight outcomes, by daily screen time use, United States, National Health Interview Survey-Teen, July 2021–December 2023. All values differed significantly from teens with less than 4 hours of daily screen time (*P* < .05).

Approximately half (50.4%) of all teens had 4 or more hours of daily screen time (Table 1). Teens with high daily screen time were more likely to have infrequent physical activity (45.6% vs 32.1%) and infrequent strength training (23.0% vs 13.3%). In addition, they were more likely to be infrequently well-rested (59.9% vs 40.1%) and to have an irregular sleep routine (49.2% vs 29.2%), as well as more likely to have weight concerns (37.8% vs 25.3%) (Figure 1).

**Table 1:** Percentage of Teenagers With ≥4 Hours of Daily Screentime Use (N = 1,952), by Study Sample Characteristic, National Health Interview Survey-Teen, United States, July 2021–December 2023^a^

Characteristic	% (95% CI)	SE	Prevalence ratio (95% CI)
**Overall**	50.4 (47.6–53.2)	1.40	—
**Age, y**			
12–14	45.6 (41.5–49.8)	2.07	0.83 (0.74–0.93)
15–17	55.0 (51.2–58.9)	1.93	1 [Reference]
**Sex**			
Male	48.3 (44.4–52.2)	1.95	0.92 (0.82–1.02)
Female	52.5 (48.5–56.5)	2.00	1 [Reference]
**Race and Hispanic origin**			
Non-Hispanic Asian	43.5 (33.4–53.9)	5.03	0.91 (0.71–1.56)
Non-Hispanic Black	60.4 (51.4–68.9)	4.30	1.26 (1.07–1.49)
Non-Hispanic White	47.9 (44.0–51.8)	1.94	1 [Reference]
Hispanic	52.8 (46.6–58.8)	3.03	1.10 (0.96–1.27)
**Urbanization level**			
Metropolitan	51.4 (48.5–54.4)	1.49	1.19 (0.99–1.42)
Nonmetropolitan	43.3 (35.8–51.0)	3.75	1 [Reference]
**Highest level of parents’ education**			
Some college or less	55.0 (50.6–59.3)	2.17	1.21 (1.09–1.35)
College degree or higher	45.2 (41.7–48.7)	1.76	1 [Reference]
**Family income as a percentage of the federal poverty level**			
<200%	51.7 (46.2–57.3)	2.77	1.04 (0.92–1.18)
≥200%	49.6 (46.3–52.9)	1.65	1 [Reference]

a All estimates are weighted to the national population.

**Table:** 

Outcome	Screen time	≥4 Hours
	<4 Hours	
Infrequent physical activity	32.1	45.6
Infrequent strength training	13.3	23.0
Infrequently well-rested	40.1	59.9
Irregular sleep routine	29.2	49.2
Weight concerns	25.3	37.8

**Figure 2:**
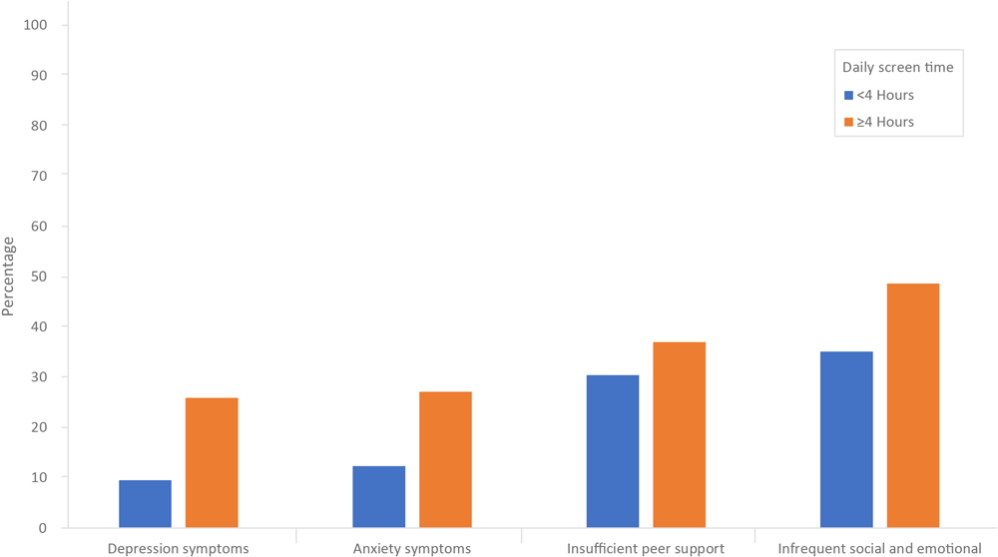
Prevalence of mental health and perceived support outcomes, by daily screen time use, United States, National Health Interview Survey-Teen, July 2021–December 2023. All values differed significantly from teens with less than 4 hours of daily screen time (*P* < .05).

Teens with high daily screen time were more likely to have depression symptoms (25.9% vs 9.5%) and anxiety symptoms (27.1% vs 12.3%) in the past 2 weeks compared with teens without high daily screen time (Figure 2). They were also more likely to report having infrequent social and emotional support (48.6% vs 35.1%) and insufficient peer support (37.0% vs 30.4%).

**Table:** 

Outcome	Screen time	≥4 Hours
	<4 Hours	
Depression symptoms	9.5	25.9
Anxiety symptoms	12.3	27.1
Insufficient peer support	30.4	37.0
Infrequent social and emotional support	35.1	48.6

In all adjusted models, with one exception, high daily screen time continued to be associated with poorer health outcomes. The adjusted analysis showed that teens with high daily screen time remained more likely to have infrequent physical activity (adjusted prevalence ratio [APR] = 1.33; 95% CI, 1.14–1.54) and infrequent strength training (APR = 1.64; 95% CI, 1.27–2.11) (Table 2). They were also more likely to be infrequently well-rested (APR = 1.45; 95% CI, 1.30–1.63) and to have weight concerns (APR = 1.42; 95% CI, 1.20–1.69), depression symptoms (APR = 2.51; 95% CI, 1.89–3.35), anxiety symptoms (APR = 2.12; 95% CI, 1.67–2.71), infrequent social and emotional support (APR = 1.29; 95% CI, 1.13–1.47), and an irregular sleep routine (APR = 1.58; 95% CI, 1.36–1.83). Although teens with high daily screen time were more likely to have insufficient peer support in unadjusted models (PR = 1.22; 95% CI, 1.05–1.42), this association was attenuated following adjustment for covariates (APR = 1.16; 95% CI, 0.99–1.35). In a sensitivity analysis, an interaction between 4 or more hours of screen time and being male was found for weight concerns (APR = 0.64; 95% CI, 0.45–0.91) and 4 or more hours of screen time and being aged 12 to 14 years for being infrequently well-rested (APR = 1.33; 95% CI, 1.06–1.67).

**Table 2:** Logistic Regression Models Presenting Associations Between ≥4 Hours of Screen Time and Health Outcomes Among Teenagers, National Health Interview Survey-Teen, United States, July 2021–December 2023^a^

Overall sample	Model 1: PR (95% CI)	Model 2: APR (95% CI)
**Physical activity**		
Infrequent physical activity	1.42 (1.22–1.66)^b^	1.33 (1.14–1.54)^b^
Infrequent strength training	1.73 (1.35–2.22)^b^	1.64 (1.27–2.11)^b^
**Sleep**		
Infrequently well-rested	1.49 (1.32–1.68)^b^	1.45 (1.30–1.63)^b^
Irregular sleep routine	1.67 (1.44–1.93)^b^	1.58 (1.36–1.83)^b^
**Weight**		
Weight concerns	1.49 (1.26–1.77)^b^	1.42 (1.20–1.69)^b^
**Mental health**		
Depression symptoms	2.74 (2.04–3.67)^b^	2.51 (1.89–3.35)^b^
Anxiety symptoms	2.21 (1.72–2.82)^b^	2.12 (1.67–2.71)^b^
**Perceived support**		
Insufficient peer support	1.22 (1.05–1.42)^c^	1.16 (0.99–1.35)
Infrequent social and emotional support	1.38 (1.20–1.59)^b^	1.29 (1.13–1.47)^b^

Abbreviations: APR, adjusted prevalence ratio; PR, prevalence ratio.

a All analyses used NHIS-Teen weighting. Model 2 was adjusted for parent-reported teen age (12–14 y, 15–17 y), sex (male, female), race or Hispanic origin (non-Hispanic Asian, non-Hispanic other/multiracial, non-Hispanic Black, non-Hispanic White, Hispanic), urbanization level (metropolitan, nonmetropolitan), parental education (some college or less, college degree or higher), and family income as a percentage of the federal poverty level (<200%, ≥200%). The reference group for all prevalence ratios was less than 4 hours of screen time.

b *P* < .001.

c *P* = .01.

## Discussion

High daily screen time was prevalent with over half of all teens having 4 or more hours of daily screen time. High screen use was consistently associated with poorer health outcomes among teens, aligning with much of the current literature. High screen time has been associated with lower rates of exercise and strength training, as well as an increased obesity risk and higher adiposity measures (25,26). High screen time has also been associated with later bedtimes, insufficient sleep duration, reduced sleep efficiency, insomnia symptoms, and excessive daytime sleepiness (27,28). Use of screens not only delays the time teens go to bed and fall asleep, but can also affect their circadian timing, sleep physiology, and alertness due to the light emitted from the screens themselves (29).

Screens can be a contributor to poorer mental health, but they also may have some value as coping mechanisms for teens who are experiencing feelings of isolation (30). Nonetheless, teens with high levels of daily screen time were more likely to report both anxiety and depression symptoms, even after adjustment for covariates, mirroring findings in recent years (13,31). Although teens have indicated that daily screen time can be used for positive purposes, like social check-ins, other uses such as accessing social media daily still have the potential to lead to social isolation and fear of missing out on activities with peers (32), with some teens acknowledging the addictive qualities of social media consumption (33). The association between screen time and social isolation and loneliness has been established in several studies (34). This study is the first to our knowledge to examine whether perceived social and emotional supports are less frequent among teens with high daily screen time. However, these findings are in parallel with previous research indicating that high social media use may be a barrier to in-person communication with friends and family (35).

Additional findings highlight associations between high screen time use and weight concerns among teens. Of note, this study did not incorporate information about teens’ actual weight but relied only on concerns about their own weight. However, several observational studies have documented that increased daily screen time is associated with greater body dissatisfaction and eating disorder symptoms, body dysmorphia, including a fear of weight gain, self-worth tied to weight, and weight-changing behaviors (36).

### Strengths and limitations

This study has several strengths. NHIS-Teen is a large and timely nationally representative survey of teens, providing sufficient sample size to explore prevalence among various subgroups. NHIS-Teen is also supplemented by data on sociodemographic characteristics provided by parents as part of the NHIS, such as health insurance and family income, which may be inaccurately reported by teens. Estimates of daily screen time from NHIS-Teen were considerably higher than those reported by parents as part of the National Survey of Children’s Health (NSCH) during the same period (37). The use of teen self-reported data can lead to a more accurate estimate of screen time use (38).

Despite these strengths, some limitations should be considered when reviewing these findings. Similar to other studies like the NSCH and YRBS, the cross-sectional nature of NHIS-Teen prevents the ability to establish causality or directionality when discussing the associations between screen time and health outcomes. Future research would benefit from a longitudinal design that could further investigate causal mechanisms. Types, content, and quality of screen usage were not accounted for and could be important contextual factors of screen time use and their impact on health, including the potential beneficial use of some screen use (39). Further, subjective measures of reported screen time and health outcomes were used instead of objective time-collecting indicators. As such, validating teen responses was not possible. Finally, despite the use of a web-based data collection tool, teens may be deliberately inaccurate in their answers due to social desirability (40) and expectations for how much screen time they should report. However, NHIS-Teen implements several quality control measures such as removing teenagers from the final data set who skipped a significant number of questions, sped through the survey, or provided nonsensical answers (resulting in approximately 0.5% of the original sample being removed).

### Implications

The American Academy of Pediatrics (AAP) rescinded their screen time guidelines in 2016 as use of screens became ubiquitous and the variety of ways to engage with screens dramatically increased; in their place was the recommendation to develop a family media plan with goals to create an appropriate balance for media time and establish consistent rules about screen use and boundaries for accessing content (41). These include parental strategies for managing their teen’s screen time as well as encouraging offline activities and hobbies. The AAP has also highlighted the role of pediatricians in promoting healthy and active living by helping teens balance media use, healthy eating, and exercise (42). Future surveys dedicated to teen health may benefit from added questions about whether family rules exist about screen use and whether conversations have occurred between teens and their doctors about healthy screen use, including the time spent on screens, devices, location of use, content viewed, and any interactions that may be occurring. They may also attempt to better understand the relationship between sleep, physical activity, and sedentary behaviors over a typical teen’s day (43).

### Conclusions

Findings from this study highlight associations between increased screen time and a variety of poorer health outcomes spanning across multiple domains, including mental health and perceived support.

## Appendix Distribution of Sociodemographic Characteristics, by Daily Screen Time, United States, National Health Interview Survey-Teen, July 2021–December 2023^a^

**Table:**

Sociodemographic characteristic	≥4 Hours, % (95% CI) (n = 987)	<4 hours, % (95% CI) (n = 965)
**Overall**	50.4 (47.6–53.2)	49.6 (46.8–52.4)
**Age, y**		
12–14	44.9 (41.0–48.8)	54.3 (50.1–58.4)
15–17	55.1 (51.2–59.0)	45.7 (41.6–49.9)
**Sex**		
Male	49.2 (45.1–53.4)	53.5 (49.5–57.4)
Female	50.8 (46.6–54.9)	46.5 (42.6–50.5)
**Race and Hispanic origin**		
Non-Hispanic Asian	3.8 (2.7–5.2)	5.1 (3.8–6.6)
Non-Hispanic Black	15.0 (11.9–18.5)	10.0 (7.3–13.2)
Non-Hispanic White	48.5 (44.5–52.5)	53.6 (49.5–57.6)
Hispanic	27.8 (24.1–31.7)	25.2 (21.4–29.3)
Non-Hispanic Other/multiracial	4.9 (3.6–6.6)	6.2 (4.2–8.7)
**Urbanization level**		
Metropolitan	88.8 (85.2–91.7)	85.1 (80.9–88.7)
Nonmetropolitan	11.2 (8.3–14.8)	14.9 (11.3–19.1)
**Highest level of parents’ education**		
Some college or less	57.0 (53.2–60.7)	47.2 (43.0–51.4)
College degree or higher	43.0 (39.3–46.8)	52.8 (48.6–57.0)
**Family income as a percentage of the federal poverty level**		
<200%	36.6 (32.6–40.7)	34.7 (30.4–39.2)
≥200%	63.4 (59.3–67.4)	65.3 (60.8–69.6)

^a^ All estimates are weighted to the national population.
